# Supplementary material for: Phenotypic screening of 1,953 FDA-approved drugs reveals 26 hits with potential for repurposing for Peyronie’s disease
Source: PLoS One. 2022 Dec 12;17(12):e0277646. doi: 10.1371/journal.pone.0277646 (PMC9744312; doi:10.1371/journal.pone.0277646)
Supplement: Attachment — Submitted filename: Response to Reviewers.docx [file pone.0277646.s002.docx]

Reviewer #1: The manuscript investigates whether phenotypic screening can be used to repurpose drugs for the treatment of Peyronie’s disease (PD). Authors have used primary fibroblasts from PD patients in a phenotypic screening assay that measures TGF-β1 induced αSMA protein synthesis. Using this screening assay, authors have screened 1,953 FDA-approved drugs and have identified 26 drugs that inhibits TGF-β1 induced αSMA protein synthesis. Moreover, the authors have also identified 10 drugs that increases TGF-β1 induced αSMA protein synthesis. The authors suggest phenotypic screening of FDA-approved drugs for PD is a viable method to predict drugs for repurposing to treat early PD. Although, the approach is interesting and supported with some experimental data, many issues remain to be addressed.

Major comments:

1. Secondary experiments like western blotting or qPCR or αSMA stress fibers by immunofluorescence for those 36 drugs that affected αSMA protein synthesis would be helpful to validate the phenotypic screening assay.

We thank the reviewer for the suggestion of these secondary assays. We expanded the chapter ‘Confirmation of hits’ and have now included a figure that uses immunocytochemistry that demonstrates the effect of the selected three hit drugs on αSMA stress fibre formation, as suggested (Fig 3). We further tested these three drugs on ECM production and cell viability (please see the point below). We used these assays to confirm the three randomly selected hit drugs that we followed up via concentration-response-curves previously. Whilst it would be interesting to follow up all 26 hits that decreased myofibroblast transformation, it is beyond the scope of this manuscript which aims to report the findings from 1,953 drugs We have previously validated the assay after it revealed drugs in a smaller screening campaign and these hits (PDE5 inhibitors and selective estrogen receptor modulators) were validated using both, in vitro and in vivo experiments, highlighting the ability of the assay to identify relevant drugs (Ilg et al, 2019). Furthermore, the combination of these two drug classes have been shown to be effective in patients with early PD in the clinic (Megson et al 2022) which further validates the applicability of our screening assay.

2. Apart from αSMA, other assays such as collagen gel contraction assay or TGF-β reporter assay using tMLC system would help to strengthen the data on myofibroblast transformation

We thank the reviewer for the suggestion and have included the outcome of an ECM production assay to further confirm the anti-myofibroblast activity of the three randomly selected hit drugs (Fig 4). As can be seen, ECM production is significantly reduced upon drug treatment, further confirming anti-myofibroblast activity of the drugs.

3. Cell proliferation assay for those selected 36 drugs would be useful to evaluate the effects of these drugs

Whilst we agree with the reviewer that it would be of interest to include data on cell proliferation, we only included the data for the three drugs that we previously confirmed via concentration response curve. We have now included a separate figure (Fig 5) to showcase that cell proliferation increases in cells treated with TGF- β1, which is reduced after treatment with the drugs. The graph depicts cells number (proliferation by proxy) derived from the nuclear stain DRAQ5 which emits at 700nm.

Minor comments:

1. The phenotypic screening assay measures the ability of the drugs to inhibit TGF-β1 induced αSMA protein synthesis but does not measures the ability of the drugs to inhibit TGF-β1 activation

The reviewer is right to point this out. Whilst we cannot claim that the drugs prevent TGF- β1 activation, we can say that the drugs inhibit TGF- β1-induced myofibroblast transformation, a downstream consequence of TGF- β1 activation. Given the agnostic nature of a phenotypic assay, it is not possible to suggest a potential mechanism of action, without target deconvolution. We have now reworded the text where this was unclear.

2. In the results section, the isolated fibroblasts were mentioned as vimentin negative and needs to be clarified. In methods, section it is written as vimentin positive.

We apologize for this mistake. The cells were desmin negative and vimentin positive. This has now been corrected in the results section. We thank the reviewer for pointing out this mistake.

3. Low serum or serum-free conditions would have helped to reduce the effects from growth factors or other protein in the serum

We agree with the reviewer on this, as this was our initial thought as well. When we developed the assay, we determined whether the use of serum free media would improve the TGF- β1-induced fold-change and/or well/well consistency. We tested serum concentrations of 10%, 1%, and 0.1% and assessed the influence on TGF- β1-induced fold-change of αSMA protein expression. No significant difference could be noted in fold-change, however, serum deprivation caused cell loss, leading to well/well inconsistency resulting in a less robust screening assay. Therefore, it was concluded that serum free conditions were not beneficial in our settings. We have now added a section in the discussion chapter to explain all optimisation steps we have taken while developing this assay.
