# Supplementary material for: Phenotypic screening of 1,953 FDA-approved drugs reveals 26 hits with potential for repurposing for Peyronie’s disease
Source: PLoS One. 2022 Dec 12;17(12):e0277646. doi: 10.1371/journal.pone.0277646 (PMC9744312; doi:10.1371/journal.pone.0277646)
Supplement: Attachment — Submitted filename: Response to reviewer.docx [file pone.0277646.s003.docx]

**Comments to the Author**

1. If the authors have adequately addressed your comments raised in a previous round of review and you feel that this manuscript is now acceptable for publication, you may indicate that here to bypass the “Comments to the Author” section, enter your conflict of interest statement in the “Confidential to Editor” section, and submit your "Accept" recommendation.

Reviewer #1: (No Response)

2. Is the manuscript technically sound, and do the data support the conclusions?

The manuscript must describe a technically sound piece of scientific research with data that supports the conclusions. Experiments must have been conducted rigorously, with appropriate controls, replication, and sample sizes. The conclusions must be drawn appropriately based on the data presented.

Reviewer #1: Partly

3. Has the statistical analysis been performed appropriately and rigorously?

Reviewer #1: Yes

4. Have the authors made all data underlying the findings in their manuscript fully available?

The [PLOS Data policy](http://www.plosone.org/static/policies.action#sharing) requires authors to make all data underlying the findings described in their manuscript fully available without restriction, with rare exception (please refer to the Data Availability Statement in the manuscript PDF file). The data should be provided as part of the manuscript or its supporting information, or deposited to a public repository. For example, in addition to summary statistics, the data points behind means, medians and variance measures should be available. If there are restrictions on publicly sharing data—e.g. participant privacy or use of data from a third party—those must be specified.

Reviewer #1: Yes

5. Is the manuscript presented in an intelligible fashion and written in standard English?

PLOS ONE does not copyedit accepted manuscripts, so the language in submitted articles must be clear, correct, and unambiguous. Any typographical or grammatical errors should be corrected at revision, so please note any specific errors here.

Reviewer #1: Yes

6. Review Comments to the Author

Please use the space provided to explain your answers to the questions above. You may also include additional comments for the author, including concerns about dual publication, research ethics, or publication ethics. (Please upload your review as an attachment if it exceeds 20,000 characters)

Reviewer #1: The authors have made considerable efforts to address some of the issues mentioned in the last review. However, there are still some major concerns that needs to be addressed.
Major comments:
1. The resolution of the images in figure 3 could have been better and quantification was not presented. Quantification of αSMA normalized to F-actin (Phalloidin) would make the data solid. Alternatively, the authors should consider either western blotting or qPCR for the three hit drugs

We thank the reviewer for the suggestion and have now included both RT-qPCR data for ACTA2 as well as Western blot images for ASMA to support the findings (new Fig 4). As can be seen in the figure, they concur with our previously reported observations.

2. The ECM production assay to validate the myofibroblast function is appreciated but it would have been much better if the authors have performed either western blotting or qPCR for Fibronectin or Collagen I/III as these methods are not dependent on cell numbers

We have now included both RT-qPCR data for FN2 and Western blot images for fibronectin to support our findings (new Fig 6). The initial ECM production assay data is still included to show the effect of the drugs on total ECM formation, without differentiating the type of matrix protein. We have included a paragraph in the discussion to explain the effects of solifenacin succinate which we believe keeps the cells in the protomyofibroblast state instead of completely preventing myofibroblast transformation.

Minor comments:

1. According to the description in the figure legend, the images were captured at 200x magnification but scale bar was not presented. The nucleus size appear different across the images. Raw images of these data would help to evaluate the data better.

We thank the reviewer for the comment. We have now included scale bars to make comparison easier. It should be noted that it has been reported that cellular size is affected in myofibroblast transformation. Myofibroblasts have been described as larger cells with more prominent Golgi apparatus and rough endoplasmic reticulum with elongated, serrated nuclei and extensive stress fibre network (PMID: 21297493, PMID: 32673537). This has now been added to the discussion.

2. The amount of ECM measured using this assay can be influenced by cell proliferation and washing steps with ammonium hydroxide.

We agree with the reviewer and have now included Western blot images for fibronectin as suggested (new Fig 6). It might not have been evident from the manuscript. but data was normalised to cell count before lysis. We have revised the methods to reflect this better (Line 217).

3. It is very hard to find a difference between the data presented in Figure 2 and figure 5 with reference to cell proliferation and cell viability as it uses the same method. The authors should have used an alternative method to assess cell proliferation.

We do agree with the reviewer that cell proliferation assay would have been nice to show one of the characteristics of transformation of fibroblasts to myofibroblasts. However, several methods of assessing cell viability only capture a fraction of it (mitochondrial activity, metabolic activity, protease activity, membrane integrity) often overlapping with cell proliferation assays (DNA synthesis, metabolic activity). In order to measure cell proliferation adequately, several different assays have to deployed. We believe this would be outside the scope of our manuscript. We have now highlighted this as a point for future research in the limitations section.
